# Recombinant collagen hydrogels induced by disulfide bonds

**DOI:** 10.1002/jbm.a.37427

**Published:** 2022-07-14

**Authors:** Jie Wang, Jinyuan Hu, Xuan Yuan, Yingnan Li, Lijun Song, Fei Xu

**Affiliations:** ^1^ Ministry of Education Key Laboratory of Industrial Biotechnology, School of Biotechnology Jiangnan University Wuxi China; ^2^ National Health Commission Key Laboratory of Parasitic Disease Control and Prevention, Jiangsu Provincial Key Laboratory on Parasite and Vector Control Technology Jiangsu Institute of Parasitic Diseases Wuxi China

**Keywords:** application, biomaterials, collagen, construction, genetic engineering, hydrogel

## Abstract

With the characteristics of low toxicity and biodegradability, recombinant collagen‐like proteins have been chemically and genetically engineered as a scaffold for cell adhesion and proliferation. However, most of the existing hydrogels crosslinked with peptides or polymers are not pure collagen, limiting their utility as biomaterials. A major roadblock in the development of biomaterials is the need for high purity collagen that can self‐assemble into hydrogels under mild conditions. In this work, we designed a recombinant protein, S‐VCL‐S, by introducing cysteine residues into the *Streptococcus pyogenes* collagen‐like protein at both the N‐and C‐termini of the collagen with a trimerization domain (V) and a collagen domain (CL). The S‐VCL‐S protein was properly folded in complete triple helices and formed self‐supporting hydrogels without polymer modifications. In addition, the introduction of cysteines was found to play a key role in the properties of the hydrogels, including their microstructure, pore size, mechanical properties, and drug release capability. Moreover, two/three‐dimensional cell‐culture assays showed that the hydrogels are noncytotoxic and can promote long‐term cell viability. This study explored a crosslinking collagen hydrogel based on disulfide bonds and provides a design strategy for collagen‐based biomaterials.

## 1 INTRODUCTION

Hydrogels are critical biological materials that gained increased attention due to their high‐water content and potential biomedical applications.^1^ Hydrogels are three‐dimensional (3D) crosslinked networks of hydrophilic polymeric materials with the ability to absorb large amounts of liquid between their polymeric chains due to their specific structures and subsequent swelling properties.^2^ Thus, tissue engineering, such as the regeneration of bone tissue, cartilage tissue, vascular system,^3^ and corneal stroma, used hydrogels. These materials have also been used as scaffold material,^4^, ^5^ cell culture matrix,^6^ drug delivery carrier,^7^, ^8^ biosensors, and H_2_O_2_ stimulus‐response.^9^, ^10^ In recent years, multilayer porous collagen and functional hydrogel composite scaffolds have been designed as drug vehicles for cancer therapy.^11^, ^12^, ^13^, ^14^, ^15^, ^16^

Collagen is the most abundant protein in mammals, accounting for 25%–30% of proteins. This protein plays a critical role in molecular and cellular interactions in the extracellular matrix to define the shape and form of tissues. Collagen consists of a supercoiled triple‐helix made from three left‐handed polyproline‐like chains twisted together into a right‐handed triple‐helix.^17^ The natural biodegradable properties of collagen make it ideal for development as a biomedical material to promote cell growth and metabolism and can be compounded with other natural polymers, inorganic or organic materials, and biomaterials. These advantages make it suitable for tissue engineering. For example, the collagen hydrogel scaffold as a tissue engineering model has been studied in cartilage differentiation in vitro, opening the prospect of clinical applications for advanced treatment systems.^11^

Moreover, collagen hydrogels have been explored in regenerating damaged corneal stroma in vivo and improving vascularization in islets.^15^, ^16^ However, natural collagen also has limitations, such as inconsistent purity and quality. It is an easy contamination source for various pathogens carried by animals. In addition, there is significant variability in hydrogel structure and mechanical properties in hydrogels prepared from animal‐extracted type I collagen, depending on the tissue source and animal age, which impact cellular behavior.

To overcome these issues, recent advances in recombinant protein production have enabled the large‐scale production of purified biological products.^18^ Therefore, extensive research has focused on producing recombinant collagen‐like proteins such as Streptococcal collagen‐like protein 2 (Scl2), initially discovered in *Streptococcus pyogenes* (*S. pyogenes*),^19^ which can be easily engineered upscaled, and purified. However, the utility of these recombinant collagen‐like proteins is hindered by the difficulty of assembling them into stable 3D bioactive hydrogels.

These proteins are comprised of a trimerization domain (V) at the N‐terminus and a collagen domain (CL), which forms a triple‐helical structure with a melting point (Tm) around 37°C.^20^, ^21^, ^22^ These collagen‐like proteins have several advantages over traditional collagen hydrogels when used for hydrogels. These are high purity and good biocompatibility, biodegradability, molecular designability, and opening an avenue for safe biomedical materials without contamination from animal‐derived diseases.^23^

The utility of the recombinant collagen‐like proteins is currently limited due to the inability of Scl proteins to assemble into stable 3D bioactive hydrogels. Bioactive hydrogels have been fabricated by combining functionalized Scl2 proteins with high molecular weight natural polymers or crosslinking agents. Such as poly (ethylene glycol) diacrylate, gelatin, fibrin, and polysaccharide‐derived polymers,^24^, ^25^, ^26^, ^27^, ^28^ and synthetic molecules, such as polymers of acrylic acid and ethylene oxide, and its derivatives. Moreover, short peptides, integrin‐binding domains, heparin‐binding domains, hyaluronic acid‐binding sequences, chondroitin sulfate binding sequences, and matrix metalloproteinases cleavage siters have been employed for the preparation of responsive hydrogels. This was done by recombinant techniques for additional bioactivity to tune the cellular response finely.^29^, ^30^, ^31^, ^32^ However, the hydrogels formed by chemical polymerization appear to retain the properties of the polymer rather than the Scl2 protein itself, such as broad tunability and mechanical properties by varying the modified polymer concentration.

Self‐assembling systems are highly desirable when compared with methods to modify polymers chemically.^33^ Thus, specific chemical modifications, including cysteine and tyrosine residues, were introduced to bacterial collagen through covalent bonding and crosslinked with ruthenium [RuI(bpy)^3^]Cl_2_, forming collagen gels.^34^ However, metal crosslinking agents with weak biocompatibility and slow degradation rates need to be supplemented in vivo,^35^, ^36^ limiting their application in clinical research as biomaterials. Therefore, much research is still needed in the field of tissue engineering to endow pure collagen with the ability to self‐assemble into hydrogels under mild conditions.

In this work, we utilized a genetic modification approach to generate collagen hydrogels by introducing cysteine residues at both the N‐and C‐termini of the *S. pyogenes* collagen‐like gene and introducing a GFPGER motif in the collagen domain for cell adhesion. The pure recombinant collagen mutants self‐assembled into hydrogels via disulfide bridge formation between the cysteines by redox reactions, eliminating the need for exogenous crosslinking agents or polymers. The structure of the collagen‐like proteins and Tm values were characterized by circular dichroism. The network morphology of the hydrogels was examined by scanning electron microscopy (SEM) imaging, and the mechanical strength of the hydrogels was characterized by microrheology. Cell‐culture and drug‐release experiments demonstrated the potential application of hydrogels as biomaterials.

## 2 MATERIALS AND METHODS

### 2.1 Molecular design of the collagen polymers

**FIGURE 1:**
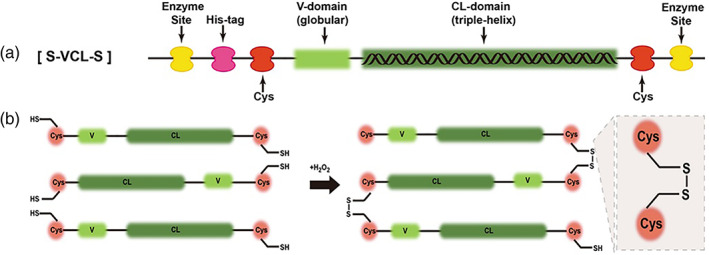
Schematic of protein design. (a) Schematic of the designed S‐VCL‐S collagen protein with Cys residues inserted at the N‐ and C‐ termini. (b) Molecular networks are induced by disulfide bonds between Cys residues

Collagen sequences containing the GFPGER region of the bioactive structure were designed. Cysteine residues were introduced at the two end sites of the collagen sequence to enable oxidative crosslinking. The Gene Designer software was used to design the target protein. The design of protein polymers VCL and S‐VCL‐S are shown in Figure 1 A‐B. Several considerations were made for optimal transcription and translation of the collagen encoding gene in *Escherichia coli* (*E. coli*) cells. These are (1) preferred codons in *E. coli* were selected. (2) High‐frequency amino acids were encoded with multiple codons. (3) Endonuclease cleaving sites were avoided in the designed sequence. (4) Complementary pairing between nucleotide sequences was avoided. (5) The GC content of the nucleotide sequence was maintained at about 50%.

### 2.2 Construction of plasmids

The *S‐VCL‐S* and *VCL* sequences were synthesized and cloned into the pET‐28a vector by GENEWIZ, Inc. (Suzhou, China) for protein expression by double enzyme digestion with *Bam*HI and *Nco*I (New England Biolabs, USA). For plasmid construction and amplification, *E. coli* DH5α cells and Luria–Bertani (LB) culture medium containing 1% tryptone, 0.5% yeast extract, and 1% sodium chloride were used.

### 2.3 Recombinant protein expression and purification

The VCL and S‐VCL‐S proteins were overexpressed in *E. coli* BL21 (DE3) cells for protein expression. A single bacterial colony was selected for culture in 4 ml LB medium, supplemented with 50 μg/ml kanamycin, at 37 °C overnight, shaking at 220 rpm. The overnight culture was diluted in fresh LB medium containing kanamycin to a ratio of 1:100 and incubated in a shaker at 220 rpm at 37°C until the optical density at 600 nm (OD600) reached 0.6–0.8. Protein expression was induced by adding isopropyl ß‐D‐1‐thiogalactopyranoside (IPTG) to a final concentration of 1 mM. The culture was further incubated for an additional 4 h. Then, the bacterial culture was centrifuged at 8000 rpm and resuspended at 4°C in binding buffer (20 mM NaPO_4_, 500 mM NaCl, 10 mM imidazole, pH 7.4) and ultra‐sonicated in ice water for 30 min. Bacterial debris was removed by centrifugation at 12,000 rpm for 20 min at 4 °C and filtered with a microporous membrane (0.45 μm). The designed proteins were purified using a 5 ml HisTrap HP column (GE Healthcare) pre‐equilibrated with the binding buffer. After washing with the binding buffer, the protein was eluted with increasing concentrations of imidazole (100, 200, 250, and 400 mM for 6 min each). The eluted proteins were desalted using a 5 ml Hitrap Desalting Column (GE Healthcare) against pure deionized water or 10 mM phosphate buffer to remove the imidazole. The protein expression was detected by sodium dodecyl sulfate‐polyacrylamide gel electrophoresis (SDS‐PAGE) analysis.

### 2.4 Mass spectroscopy

Matrix‐assisted laser desorption ionization (MALDI) mass spectrometer was acquired by matrix‐assisted laser desorption ionization time‐of‐flight (MALDI‐TOF)/TOF (Bruker Daltonics Corporation, Billerica, MA, USA) equipped with a 355 nm Smart beam II: laser with a 450 ns delay pulsed ion extraction with an accelerating voltage of 19.5 kV. The global attenuator offset of the laser is 30% using a standard linear‐positive (LP) 5 ~ 50 kDa method provided by the software. α‐cyano‐4‐hydroxycinnamic acid (CHCA) was purchased from Bruker (Billerica, MA, USA). Acetonitrile, trifluoroacetic acid (TFA), and methanol (HPLC grade) were obtained from Sigma. (Sigma‐Aldrich LLC, USA). Purified water was prepared by a Milli‐Q apparatus (Millipore, Bedford, MA, USA). The analysis procedure is described as follows: 1 μl of the VCL and S‐VCL‐S proteins solution dissolved in purified water (2 μg/μL) was deposited on a 384‐spot stainless steel MALDI plate. Then, they were mixed with 1 μl of CHCA (5 μg/μL) dissolved in acetonitrile/H_2_O (30%, V/V) and 0.1% TFA. After drying under ambient conditions, the samples were subjected to analysis on MALDI‐TOF/TOF MS.

### 2.5 Circular dichroism spectroscopy

Far‐UV circular dichroism (CD) spectra were acquired using an Applied Photophysics Chirascan with a Peltier temperature controller (Applied Photophysics Ltd, Britain). An equivalent of 1 mg/ml collagen solution in 10 mM phosphate buffer, pH 7.4 in 1 mm path length quartz cuvettes (Model 110‐OS, Hellma, USA) were equilibrated at 4°C for at least 24 h before measurement. Scans were taken from 190 to 260 nm in 1 nm steps with a 5 s averaging time. The melting experiments were performed from 4 to 80°C with a temperature increase of 1.0°C every 6 min and monitored at 220 nm. The samples were equilibrated for at least 8 s at each temperature. The melting points (T_m_) were obtained from the first derivative of the melting curves.

### 2.6 Formation of cysteine‐based disulfide bond crosslinks

To form the disulfide bond crosslinks, 90 μl of 2%–4% (wt/vol) S‐VCL‐S solution was added to a glass tube. Then, 10 μl of hydrogen peroxide (H_2_O_2_) (0.5%–1%) was added, mixed gently, and incubated in a water bath at 37°C for 30 min. In the control group, 10 μl of H_2_O_2_ was added directly to 90 μl of 2%–4% (wt/vol) VCL and incubated at 37°C for 30 min and then left at 4°C for at least 7 days. After gel formation, the glass tube was removed and placed upside down. If the polymer molecules were crosslinked to form a hydrogel, their mobility was limited and remained at the bottom of the glass tube. However, gel formation could be detected when it flowed down the tube wall. The observations were recorded with an ordinary photographic camera.

### 2.7 Microrheology

Soft materials, such as gel solutions, which have complex structures with characteristic length and time scales, can be characterized by the stress relaxation modulus, G(ϖ). Herein, we added concentrated suspensions of 1.0 μm diameter fluorescence polystyrene beads (Invitrogen, USA) to the protein samples at 2% and 4% (wt/vol). The fluorescence of the beads with an appropriate distribution density of more than 30 particles/field of view was examined by inverted microscopy (Leica, Germany). The mean square displacement (MSD) was determined as a function of time using a CCD camera (QCAM QImaging, CAN) by recording, analyzing, and imaging the trajectories of the 1.0 μm fluorescent polystyrene beads embedded in the protein samples.^37^, ^38^ The location of specific beads was determined by recordings composed of about 250 images of the trajectories analyzed by the IDL image analysis software. MSD was determined as a function of lag time, and G' and G" were determined based on the Generalized Stokes‐Einstein correlation following the methods reported by Mason et al.^39^

### 2.8 Scanning electron microscopy

The 4% (wt/vol) VCL and S‐VCL‐S proteins solution were mixed with 0.1% (wt/vol) H_2_O_2_. The hydrogels were then lyophilized with a FreeZone Plus 6 L cascade console freeze‐dry system (Labconco, Kansas City, MO, USA). The lyophilized hydrogels were sputter‐coated with a thin layer of a palladium/gold alloy to improve the surface conductivity for SEM. Images of the morphology and microstructure of the hydrogels were acquired using a Hitachi SU1510 SEM (Tokyo, Japan) with an acceleration voltage of 5 kV.

### 2.9 2D/3D cell culture on hydrogels

Mouse osteoblast cells (MC3T3‐E1 Subclone 14) were purchased from the Chinese Academy of Sciences in an alpha‐MEM medium containing 10% fetal bovine serum in an incubator at 37°C with 5% CO_2_. Cells were passaged when confluency reached 90%. After the confluency of the cells reached 100%, the medium was replaced with a complete growth medium containing ribonucleosides, deoxyribonucleosides, 2 mM L‐glutamine, and 1 mM sodium pyruvate but without ascorbic acid (GIBCO, Custom Product, Catalog No. A1049001) and was changed on the following day.

The lyophilized protein was solubilized in the culture medium to a final 4% (wt/vol) concentration and filtered using a 0.22‐μm filter membrane. Then, 10 μl of 0.5% H_2_O_2_ was mixed with 90 μl of the protein solution. A volume of 100 μl was added to a 96‐well plate and incubated at 37°C for 30 min for coagulation. The thickness of the formed gel was 2 mm. After the gel was solidified, 100 μl of the culture medium was added to the plate. The gel was rinsed three times to remove residual H_2_O_2_.

### 2.10 Live–dead assay

Calcein‐AM has very low toxicity, emits a strong green fluorescence signal after hydrolysis by lactonase, and can be used to observe the morphology of living cells. To prepare the live/dead working solution, 4 μl of ethidium homodimer‐1 and 1 μl of Calcein‐AM (MedChemExpress, China, Cat.HY‐D0041) were mixed with 2 ml of phosphate‐buffered saline (PBS). Then, 0.1 ml of live/dead working solution was added to the 96‐well plate created in the previous section and incubated at 37°C in the dark for 30 min. After two PBS washes, samples were analyzed by confocal microscopy (100x, Leica; Deerfield, IL, USA) equipped with a fluorescence light source. A commercial polystyrene‐coated cell culture plate was used as a control.

### 2.11 Detection of cell proliferation

According to the manufacturer's instructions, cell viability was assessed using a CCK8 assay (Cell Counting Kit‐8, Japan, ck04). 100 μl of cells solution was added to the 96‐well plate and incubated at 37°C with 5% CO_2_. A volume of 10 μl of CCK‐8 solution was placed into a 96‐well tissue culture plate. The plates were then incubated at 37°C for 1 h. The OD at 450 nm was measured using a microplate reader (Thermo MK3). The OD represented the relative number of viable cells. The cell viability was calculated using the following formula: Cell viability = test (concentration/control × 100)%.

### 2.12 Hydrogel encapsulation and drug release

Rhodamine B (Macklin, China, Cat. R817226) is a hydrophilic fluorescent dye with strong polarity and is widely used in controlled release research as a model drug. Rhodamine B was encapsulated by the hydrogels and eventually released into the solution. For these experiments, 5 μl Rhodamine B (1 mg/ml) was added to 90 μl of 4% (wt/vol) protein solution and mixed gently. Then, 5 μl of 0.1% H_2_O_2_ was added to the mixture, plated to a 96‐well plate, and incubated at 4°C for at least 7 days. After the gel formation, PBS (200 μl) was added to the wells, and consequently, Rhodamine B was released. The content of Rhodamine B in the solution was detected at 0, 2, 4, 6, 8, 10, 12, 24, 36, 48, and 72 h. For sampling and detection, 10 μl of the solution was collected at each time point, and an equivalent volume of PBS was replenished. The solution to be tested was diluted using fresh PBS to the ratio of 1:20. The fluorescence signal was detected using the SPECTROMAX I3 multi‐functional enzyme reader with excitation at 553 nm and recording the emission at 627 nm (Molecular Devices, LLC, San Jose, CA, USA).

A standard curve was generated by weighing out 10 mg of Rhodamine B and dissolving it in PBS to a 2 mg/ml concentration. This stock solution was then serially diluted to 0.1, 0.2, 0.4, 0.6, 0.8, and 1 mg/ml. The standard curve of the fluorescence signal of Rhodamine B against the concentration was generated by detection of the emission fluorescence value at 627 nm under an excitation wavelength of 553 nm on a microplate reader.

### 2.13 Statistical analysis

Data including cell viability are reported as mean ± standard error of the mean (*n* = 3). Comparison of sample means among multiple groups was performed by one‐way analysis of variance, and pairwise comparisons of sample means between groups were performed by Tukey's post hoc test (SPSS 20.0 software). *p*‐value < .05 was defined as significant.

## 3 RESULTS

### 3.1 Design of proteins

The sequence encoding S‐VCL‐S was designed to contain a globular domain V and a triple‐helical domain of sequence Gly‐Xaa‐Yaa CL, derived from *S. pyogenes* Scl2 sequences. A GPS triplet was inserted at the N and C‐termini of the CL domain, while cysteines were inserted at the N and C‐termini of the VCL domain. The designed protein sequences are shown in Figure 1. The S‐VCL‐S contained an N‐terminal His_6_ tag for purification (Figure 1A). To obtain hydrogels, H_2_O_2_ was added to induce the formation of intermolecular disulfide bonds between the inserted cysteines (Figure 1B). VCL was also tested as a negative control, characterized by Brodsky et al.^40^

### 3.2 Expression and purification of the designed collagen proteins

#### 3.2.1 Plasmid construction

**FIGURE 2:**
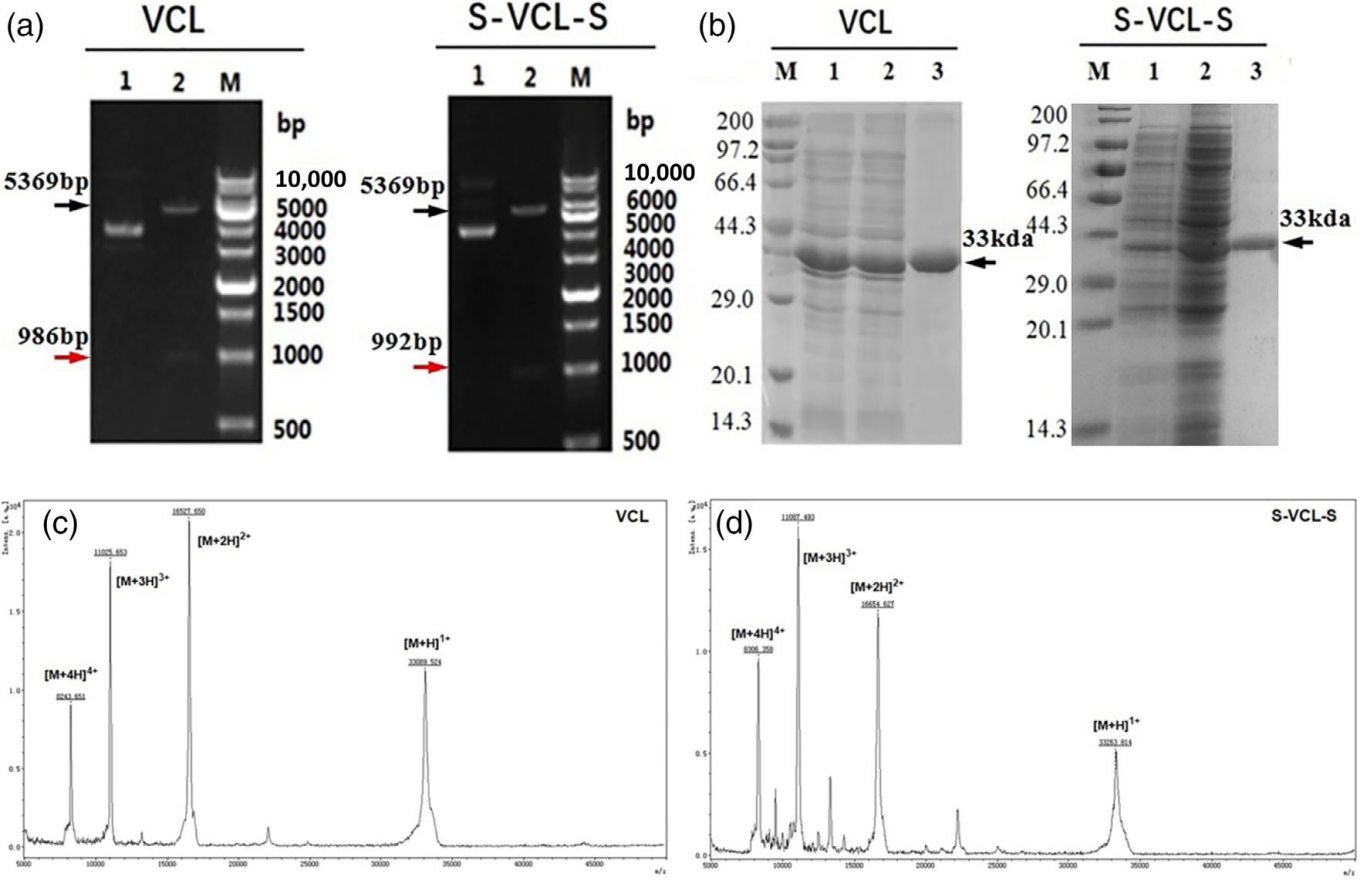
(a) The length of the nucleotide fragments after digesting the recombinant plasmids with *Bam*HI and *Nco* I enzymes. M represents the DNA marker; lane 1 shows the intact pET‐28a‐VCL and pET‐28a‐S‐VCL‐S constructs, while lane 2 shows the same constructs after double digestion by *Bam*HI and *Nco* I. (b) Molecular weights of the proteins. SDS‐PAGE analysis of the purified collagen proteins, VCL and S‐VCL‐S; M shows standard protein marker; lanes 1 and 2 show protein expression before purification of VCL and S‐VCL‐S; lane 3 shows VCL and S‐VCL‐S after purification. (c and d) Matrix‐assisted laser desorption ionization time‐of‐flight (MALDI‐TOF) mass spectrometry to characterize the molecular weights of VCL and S‐VCL‐S proteins. The four peaks are the molecular ion peaks with single, double, triple, and quadruple charges, respectively (right to left)

The pET‐28a‐VCL and pET‐28a‐S‐VCL‐S vectors were verified by double digestion using *Bam*HI and *Nco*I (Figure 2A). The length of the digested fragments of VCL and S‐VCL‐S were 986 and 992 bp, respectively, which was consistent with the designed length. The verified recombinant plasmids were transformed into *E. coli* BL21 (DE3) cells to express the designed proteins. The amino acid sequences of the designed collagen proteins are provided in the Supporting Information (Table S1).

#### 3.2.2 Protein expression and purification

The collagen proteins, VCL and S‐VCL‐S, were overexpressed in *E. coli* BL21(DE3) cells and purified by the HisTrap HP column. The molecular weight and purity were verified with SDS‐PAGE analysis (Figure 2B) and MALDI‐TOF/TOF (Figure S1). MALDI‐TOF mass spectrometry was carried out to characterize the molecular weights of VCL and S‐VCL‐S proteins. The four peaks are the molecular ion peaks with single, double, triple, and quadruple charges, respectively (Figure 2C,D: right to left). The experimental molecular weights of VCL and S‐VCL‐S were 33.127 and 33.336 kDa, respectively. The errors between the measured and theoretical values were within 0.05% for both proteins (Table S2).

### 3.3 Secondary‐structure characterization and thermal stability

**FIGURE 3:**
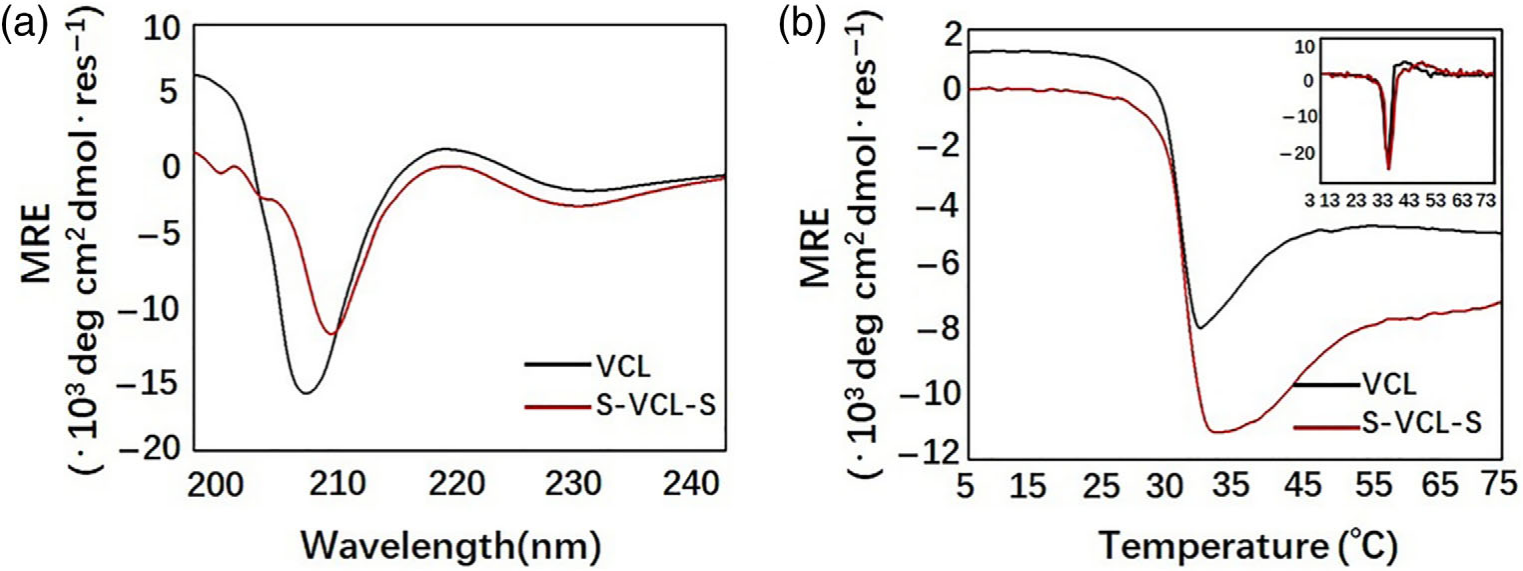
CD characterization of the designed proteins. (a) Far‐UV CD spectra of the collagen proteins and (b) Thermal denaturation profiles of VCL and S‐VCL‐S at 1 mg/mL in 10 mM phosphate buffer (pH 7.4)

To test whether the triple‐helical domain of S‐VCL‐S has the proper structure, we characterized the secondary structure of the protein with CD spectroscopy on assemblies at a low concentration (1 mg/ml) for optical transparency. The CD spectrum from 240 to 200 nm of S‐VCL‐S showed typical triple‐helical spectra with a positive peak at 220 nm and a minimum at 210 nm (Figure 3A). The ellipticity difference between both proteins was small at 220 nm. The mean residue ellipticity value of S‐VCL‐S at 220 nm was closer to zero than VCL (Figure 3A). These results confirmed that α‐helices and triple helices were similarly formed for the S‐VCL‐S protein to VCL.

The thermal stability of S‐VCL‐S was determined by monitoring the intensity of the triple helix maximum (220 nm) at increasing temperatures at a rate of 0.1°C/min. The melting transitions of the S‐VCL‐S protein showed a sigmoidal shape, resembling collagen melting curves, and were consistent with VCL. The T_m_ of S‐VCL‐S was around 37°C (Figure 3B), consistent with the negative control (VCL) observed by Brodsky et al.^41^ Notably, the protein with the cysteine residues was as stable as the native VCL, suggesting that adding cysteine residues to VCL may not disturb the formation of the secondary structures and the stability of the protein.

### 3.4 Gelation

**FIGURE 4:**
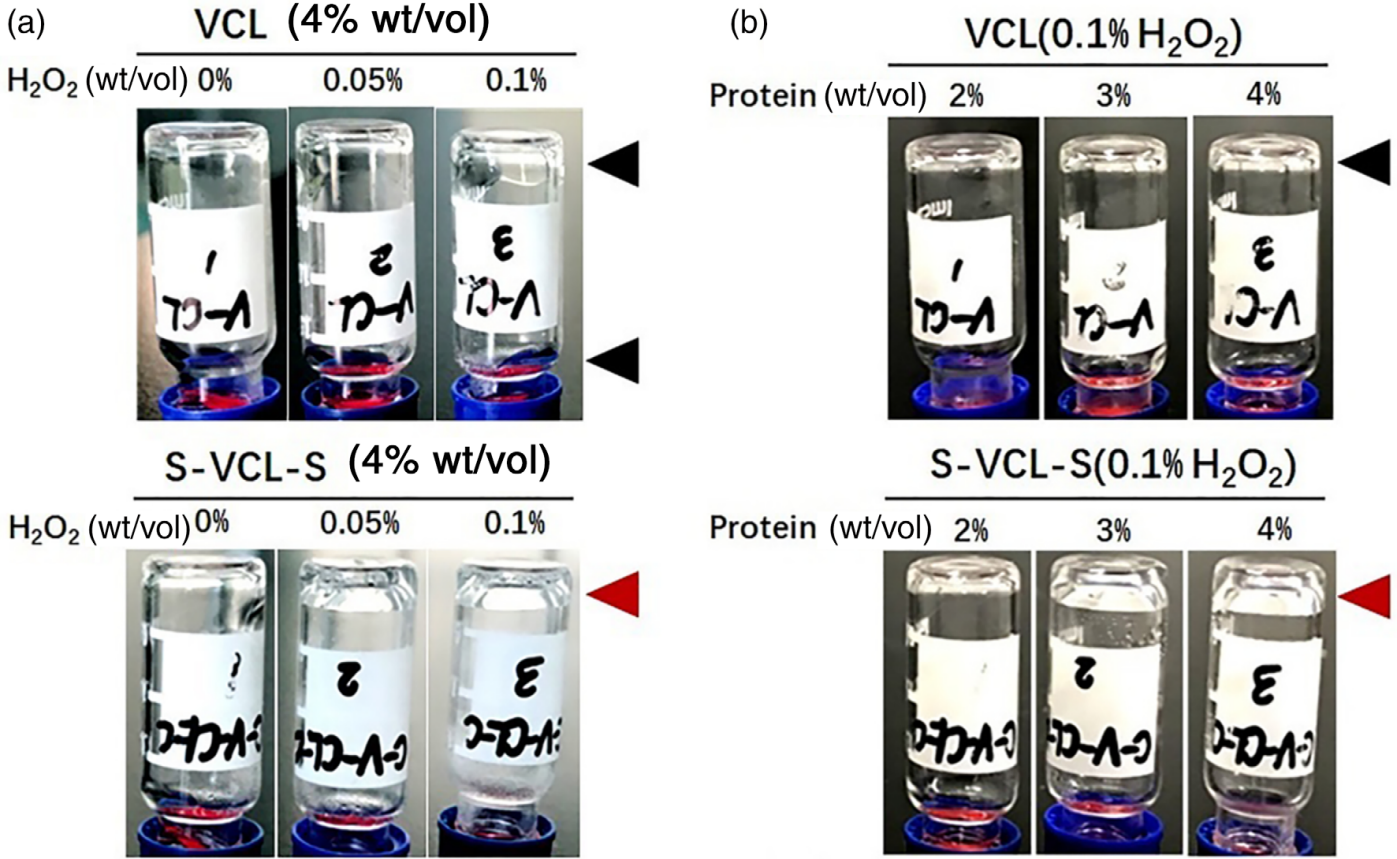
(a) Tube inversion test of hydrogel formation under various S‐VCL‐S and H_2_O_2_ concentrations. Vials containing 2%, 3%, and 4% (wt/vol) S‐VCL‐S with 0.1% H_2_O_2_ (top) or 0, 0.05, and 0.1% H_2_O_2_ with 4% S‐VCL‐S (bottom) were incubated at 37°C for 30 min and inverted for image collection. (b) Test of formation of self‐supporting hydrogels under different VCL protein and H_2_O_2_ concentrations. The concentrations tested were the same as in A

To investigate the response of the S‐VCL‐S protein to oxidative environments and its ability to form hydrogels, an inversion test was performed to characterize the hydrogels' properties using H_2_O_2_ as an oxidant. Two parameters, the concentration of protein and H_2_O_2,_ were optimized to induce hydrogel formation at 37°C (Figure 4).

We first fixed the H_2_O_2_ concentration at 0.1%, previously used for oxidative crosslinking,^42^ and varied protein concentration. Under low S‐VCL‐S concentrations (2%, wt/vol), 0.1% H_2_O_2_ could not induce hydrogel transformation, indicating that the protein concentration was unsuitable for a network of interlinking gels. When the concentration of S‐VCL‐S increased to 3%, the weak hydrogel was formed; at 4% S‐VCL‐S, it formed a stable hydrogel (Figure 4A). In the absence of H_2_O_2_, S‐VCL‐S protein remained a solution at all tested concentrations. These results showed that the S‐VCL‐S protein responds to oxidative environments to form a hydrogel network.

To reduce the influence of H_2_O_2_ on the cell experiment and further optimize hydrogel formation, the S‐VCL‐S protein was mixed with 0.05%–0.1% H_2_O_2_ at 37°C. Notably, the S‐VCL‐S protein formed a weaker hydrogel at 0.05% H_2_O_2_ than 0.1% H_2_O_2_. These findings suggested that the higher the protein and H_2_O_2_ concentration, the easier the gel formation. As a result, all subsequent testing was conducted with 4% protein solution and 0.1% H_2_O_2_.

Notably, VCL could not be oxidized by H_2_O_2_ at any concentration and remained as a solution even at concentrations up to 4% (wt/vol) (Figure 4B). These results indicated that the disulfide bonds between cysteines were crucial for hydrogel formation.

### 3.5 Microrheology characterization

**FIGURE 5:**
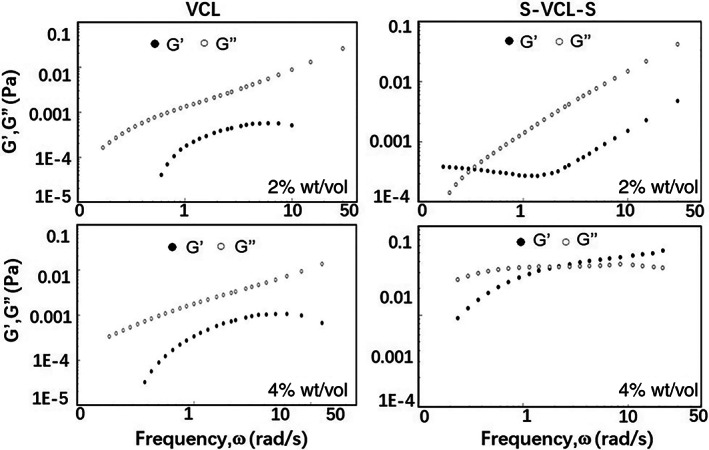
Storage and loss moduli, G' and G", of the VCL (right) and S‐VCL‐S (left) hydrogels at 2 and 4% (wt/vol) in 10 mM PBS (pH 7.4). (Above) 2% wt/vol; (below) 4% wt/vol. PBS, phosphate‐buffered saline

To further confirm the formation of the hydrogel, microrheology was used to measure the storage modulus (G') and loss modulus (G") of the hydrogel at different concentrations. S‐VCL‐S remained a solution with G">G' at low concentration (2%, wt/vol). Conversely, at a high concentration (4%, wt/vol) beyond the sol–gel transition point, S‐VCL‐S formed a soft gel with G" < G' at 3.682 rad/s. The negative control, VCL, remained as a solution at both concentrations (Figure 5). These observations were consistent with those of the tube inversion experiments.

### 3.6 Microstructures of collagen hydrogels

**FIGURE 6:**
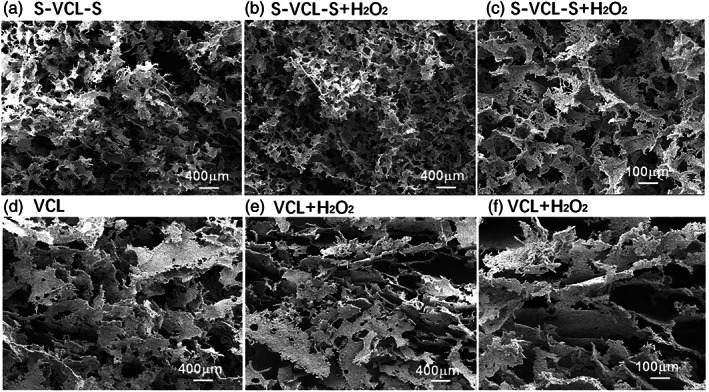
SEM micrographs of the collagen‐like proteins before and after the addition of H_2_O_2_. (a–c) S‐VCL‐S and (d–f) VCL. Scale bar = 400 μm for a, b, d, and e and 100 μm for c and f. The concentration of VCL and S‐VCL‐S is 4% (wt/vol) with PBS or without 0.1% (wt/vol) H_2_O_2_. SEM, scanning electron microscopy

To verify its effect on the microstructure of the recombinant collagen hydrogels, H_2_O_2_ was added to S‐VCL‐S, and the porous microstructures were analyzed by SEM (Figure 6). The S‐VCL‐S and VCL proteins were lyophilized before and after the addition of H_2_O_2_ and imaged with SEM. After lyophilization, both S‐VCL‐S and VCL, without H_2_O_2_, formed a loose pore structure (Figure 6A,D). After the addition of H_2_O_2_, S‐VCL‐S formed a relatively dense pore structure (Figure 6B,C). However, no change was detected for VCL under the same conditions (Figure 6D–F). These results showed that the microstructure of S‐VCL‐S hydrogels with H_2_O_2_ had a smaller pore size than those without H_2_O_2_.

### 3.7 Cell cytotoxicity and spreading assay in 2D cell culture

**FIGURE 7:**
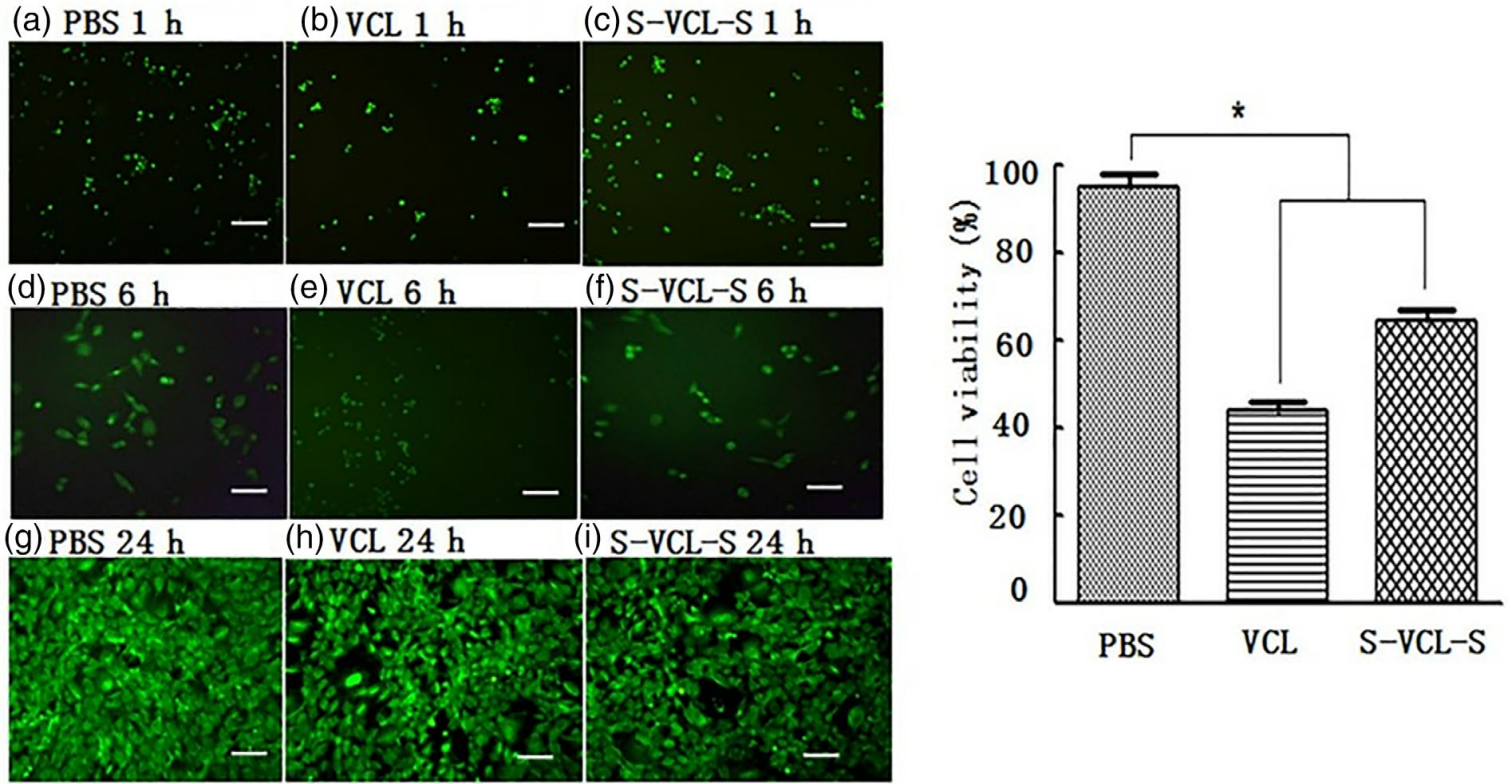
(Left) Cell viability of MC3T3‐E1 on the hydrogel surface (living cells are labeled with green fluorescence by Calcein AM) (a–f). (Right) Cell viability of MC3T3‐E1 on the surface of different types of hydrogels after 24 h. Significantly different from the corresponding control, *p* < .05 (*n* = 3)

To investigate whether oxidatively crosslinked S‐VCL‐S hydrogels can support cell growth, MC3T3‐E1 were seeded on the surface of the hydrogels to detect cell adhesion and proliferation. As shown in Figure 7, Calcein AM fluorescence was observed 1, 6, and 24 h after cell inoculation. The control and oxidatively‐crosslinked S‐VCL‐S hydrogels stretched and attached to the surface of the plate, indicating that the hydrogel material could be used for cell adhesion. After 1 day post‐inoculation on the material's surface, the number of cells on the S‐VCL‐S hydrogels and VCL protein was 69.15 ± 1.51 and 43.32% ± 1.83 of that of the control group, respectively, as estimated by the CCK‐8 Kit (Figure 7). Compared to the cells in 96‐well plates with only PBS, the cell growth on S‐VCL‐S was impacted by the uneven surface of the hydrogel. In the cell plate with VCL protein, residual H_2_O_2_ had a stimulating effect on cell growth. The cell viability of the two experimental and control groups was significantly different (*p* < 0.05). These results indicated that the collagen hydrogels were not cytotoxic.

### 3.8 Cell cytotoxicity and spreading assay in 3D cell culture

**FIGURE 8:**
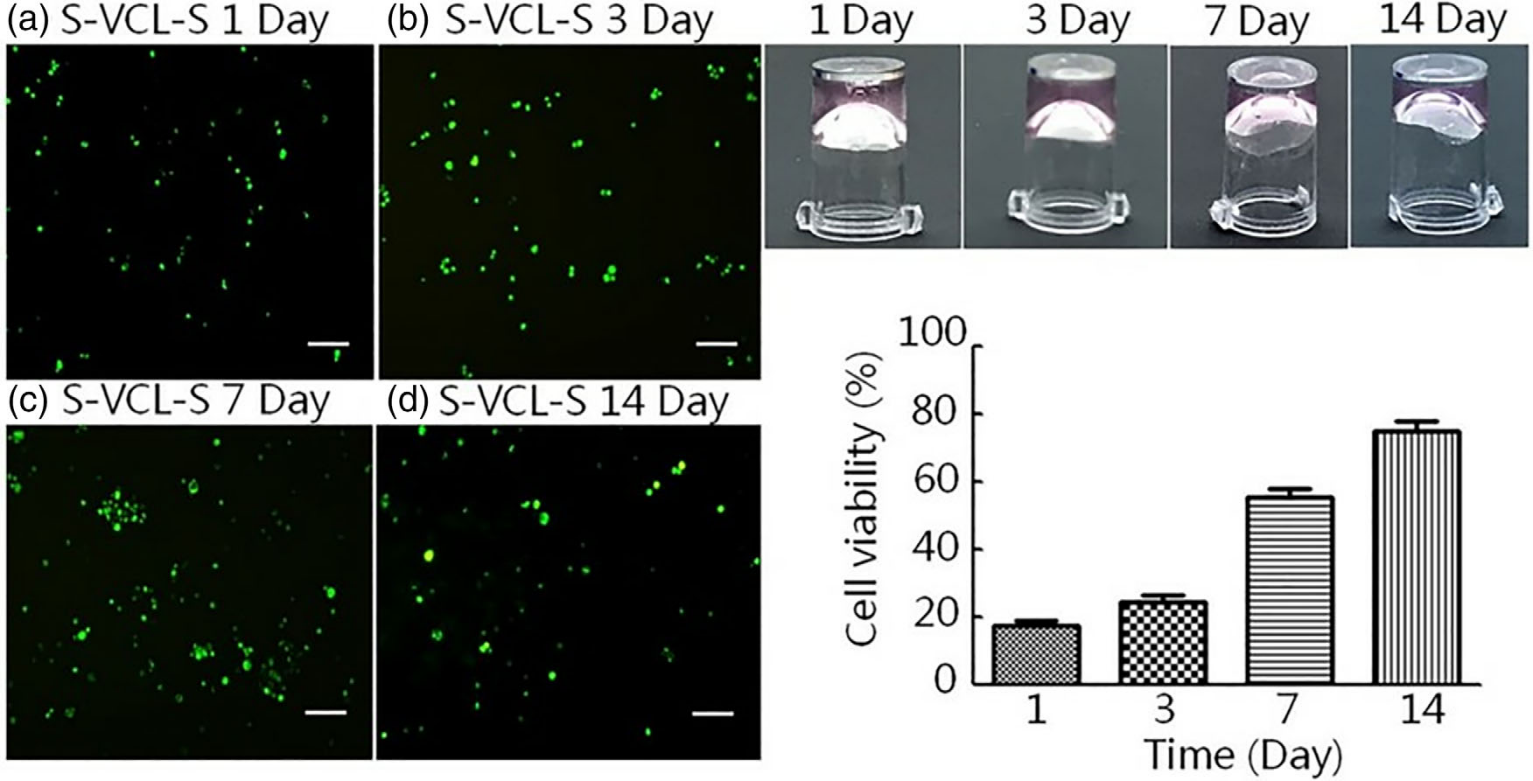
(Left) Live/dead staining for 3D cell culture on days 1, 3, 7, and 14 (a–d). Scale bar: 100 μm. Living cells were labeled with green fluorescence by Calcein AM. The number of green fluorescence‐positive cells was determined. (Right) Cell spreading on plates coated with the designed collagens. Cells were seeded and spread on tissue culture plates coated with S‐VCL‐S in 4% (wt/vol) for 1 day to 2 weeks. The observations were recorded by taking photos using a camera (top), and the cell viability of MC3T3‐E1 in S‐VCL‐S hydrogels was calculated (bottom). Statistically significantly differences from the cell viability at 1‐, 3‐, 7‐, and 14‐days (*p* < .05) (*n* = 3)

To better simulate in vivo cell growth and further verify whether the hydrogels could support cell growth in a 3D environment, the MC3T3‐E1 were encapsulated inside the hydrogel for culture. Live/dead staining was performed to determine cell survival in the hydrogel. The growth status of the hydrogel‐wrapped cells was detected after 1, 3, 7, and 14 days. As shown in Figure 8, the cells were packaged inside the hydrogel and survived for up to 14 days, with no apparent cell death. Within 24 h, cells had spread on the S‐VCL‐S hydrogels with the expected cell morphology and distribution density (Figure 8). The cell viability was measured by a CCK‐8 Kit with detection at 450 nm (Figure 8). Cell viability in the 3D cell culture at 1‐, 3‐, and 7‐days post‐inoculation was about 18.23 ± 1.17%, 25.42 ± 1.75%, and 58.61 ± 1.61%, which is less than the control, respectively. The cell viability gradually increased after 2 weeks to 78.66 ± 2.12%. There were significant differences in the cell viability at 1‐, 3‐, 7‐, and 14‐days (*p* < .05). These results suggested that the designed recombinant collagen can form 3D noncytotoxic hydrogels and support cell growth.

### 3.9 Redox sensitivity of the hydrogels

**FIGURE 9:**
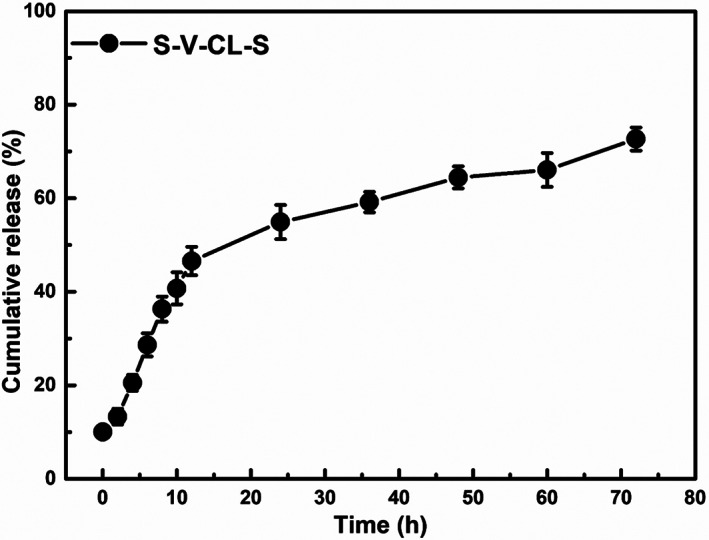
Sustained release of Rhodamine B from the hydrogels. A solution of 50 μg/ml Rhodamine B, 0.05% (wt/vol) H_2_O_2_, and 4% (wt/vol) S‐VCL‐S protein were mixed to form hydrogels at 37°C on a 96‐well plate. Rhodamine B release was initiated by dropping PBS onto the hydrogel surface. PBS, phosphate‐buffered saline

To observe the effect of hydrogels on the drug release rate under different environmental conditions, hydrogel‐encapsulated model drugs (Rhodamine B) were used to determine the response of the hydrogels to the environment and observe the effect of hydrogels on the drug release rate. As shown in Figure 9, the cumulative release efficiency of Rhodamine B from S‐VCL‐S hydrogels was determined. The S‐VCL‐S hydrogels released 72.05% ± 3.32% of the Rhodamine B after 72 h in the release buffer, gradually released into the solution. These results demonstrated that the S‐VCL‐S collagen hydrogels could act as drug delivery platforms, which could meet the needs of biomaterials.

## 4 DISCUSSION

Recombinant collagen‐like proteins are now regarded as an ideal source for novel biomaterial products. For example, Scl2 can be easily fermented and purified on a large scale,^20^ with consistent and well‐defined production processes essential for pharmaceutical purposes. However, Scl2 cannot self‐assemble into gels as blank template protein, and the use of polymers and metal ions is currently not available in clinical practice. This study explored a method of crosslinking collagen hydrogels based on disulfide bonds. Its network morphology was examined by SEM and characterized with microrheology. The results demonstrated that hydrogels may have potential application as biomaterials and suggested that disulfide bonds can promote the crosslinking of collagen molecules to form hydrogels.

Self‐assembling systems are highly desirable compared with methods to modify polymers chemically.^33^ Thus, we designed a new recombinant protein containing the complete globular domain V and a triple‐helical domain of sequence Gly‐Xaa‐Yaa CL. Two cysteines were inserted at the N‐ and C‐termini of the VCL domain. The resulting construct was successfully expressed and purified from a bacterial system with similar thermal stability and secondary structure of the parenting Scl2 previously published by Brodsky et al.,^41^ with the added advantage of hydrogel formation under oxidative conditions. Previous studies have focused on specific chemical modifications, e.g., cysteine and tyrosine residues, for covalent bonding and crosslinking with ruthenium [RuI(bpy)^3^]Cl_2_, that also successfully form collagen gels.^34^ However, metal crosslinking agents with weak biocompatibility and slow degradation rates need to be supplemented in vivo,^35^, ^36^ limiting their applicability in clinical research as biomaterials. In contrast, the novel S‐VCL‐S construct here presented can be from a hydrogel structure under oxidative environments.

In the presence of 0.1% H_2_O_2_, a 4% (wt/vol) solution S‐VCL‐S self‐assembles into a visible hydrogel, which can be checked macroscopically. This was further confirmed by microrheology but with a much lower concentration so samples could be easily injected into the capillary space. Hence, the values reported for the elastic moduli in the microrheology experiments were smaller than those expected from the tube‐inverting experiments. Nonetheless, the formation of a hydrogel matrix was further validated and characterized by electron microscopy. In addition, the concentrations of H_2_O_2_ and the S‐VCL‐S protein were found to play a key role in the hydrogel properties. A translucent gel could be formed under 4% (wt/vol) S‐VCL‐S and 0.1% (wt/vol) H_2_O_2_. However, if the protein concentration is less than 4% and H_2_O_2_ is less than 0.05%, hydrogels could not be formed.

Biomaterials are frequently used as cell culture matrix^6^ or drug delivery carrier.^7^, ^8^ S‐VCL‐S hydrogels could sustain MC3T3‐E1 adhesion and growth. Cell growth seemed slightly impaired compared to controls, but this was true for both Scl2 protein and S‐VCL‐S. Ramshaw et al. observed that the cell viability of cells grown on Scl2 collagen proteins was lower than natural collagen.^20^ A similar phenomenon was observed in our study, likely due to the impact of residual H_2_O_2_ levels on cell proliferation. In a 3D cell culture model, MC3T3‐E1 could be encapsulated in S‐VCL‐S hydrogels with minimal impact on cell viability. Others have shown a reduction in cell spreading with decreasing substrate stiffness^43^; and again, Ramshaw et al. reported decreased cell viability on Scl2 compared to natural collagen control.^20^ Therefore, a decrease in cell viability during culture in S‐VCL‐S hydrogels was expected. Nonetheless, it is encouraging to see that cell viability improves over time and is almost fully recovered 14 days after inoculation. Despite limited growth space, the S‐VCL‐S hydrogels support cell survival and proliferation.

S‐VCL‐S hydrogels could also act as drug carriers. Oxidative‐crosslink S‐VCL‐S hydrogels could gradually release Rhodamine B for 72 h into the solution. Such release rates appear slower than some compounds, such as S4E8C hydrogels. However, it was higher than SE8C and S2E8C hydrogels, which might be related to their microstructures.^42^

The ratio of Cys to the total number of residues is positively correlated with gel strength. Gel strength increases with the higher ratios, from 0.3% to 26.7%, whereas it decreases with lower ratios.^34^, ^42^, ^44^, ^45^ These results suggested that disulfide bonds can promote the crosslinking of collagen molecules to form hydrogels. In addition, the mechanical strength of the hydrogel increases with an increase in the crosslinking degree of disulfide bonds. Furthermore, gel stiffness increased according to the number of Cys residues; however, the stiffness reached a limit when the ratio of Cys to the total residue number was 20% or higher.^45^

This study provides a means of chemical crosslinking collagen hydrogels based on disulfide bonds, which can be further optimized by adjusting the amount of cysteine to control the properties of the materials. This work could find broader applications in drug delivery and tissue engineering in the future.

## 5 CONCLUSION

In this study, we developed a recombinant collagen‐like protein, S‐VCL‐S, where Cys residues were inducted into the N and C‐termini of the VCL domain. The S‐VCL‐S reacted with H_2_O_2_, a mild redox agent, to generate covalently crosslinked hydrogel through disulfide bridges between the cysteines by the oxidation reaction. The 2D/3D cell‐culture results showed that the hydrogels are noncytotoxic and can promote long‐term cell viability. The hydrogels have typical mesh‐like structure characteristics, significantly improve drug release capability, and have broad application prospects. It can sustain cell growth on a 2D and 3D cell culture model, sufficient for long‐term cell viability. This work lays a foundation for the future design of hydrogels to enhance their mechanical properties by adjusting the length of the collagen domain and the location and density of cysteine residues and provides a perspective for studies on new drug sustained‐release carriers for clinical applications.

## CONFLICT OF INTERESTS

The authors declare that they have no conflict of interest.

## Supporting information


**Figure S1** Supporting Information

**Supplemental Table 1** The designed sequence of VCL and S‐VCL‐S

**Supplemental Table 2** The theoretical and measured molecular weight of protein

**Supplemental Table 3** Comparison of cell viability at 1 day among two experimental groups and the control group
**Supplemental Table 4**. Comparison of cell viability at 1‐, 3‐, 7‐, and 14 days in experimental groups

## Data Availability

Data are available on request from the authors.
