# Supplementary figures and images for: Recombinant collagen hydrogels induced by disulfide bonds
Source: J Biomed Mater Res A. 2022 Jul 14;110(11):1774–85. doi: 10.1002/jbm.a.37427 (PMC9544300; doi:10.1002/jbm.a.37427)

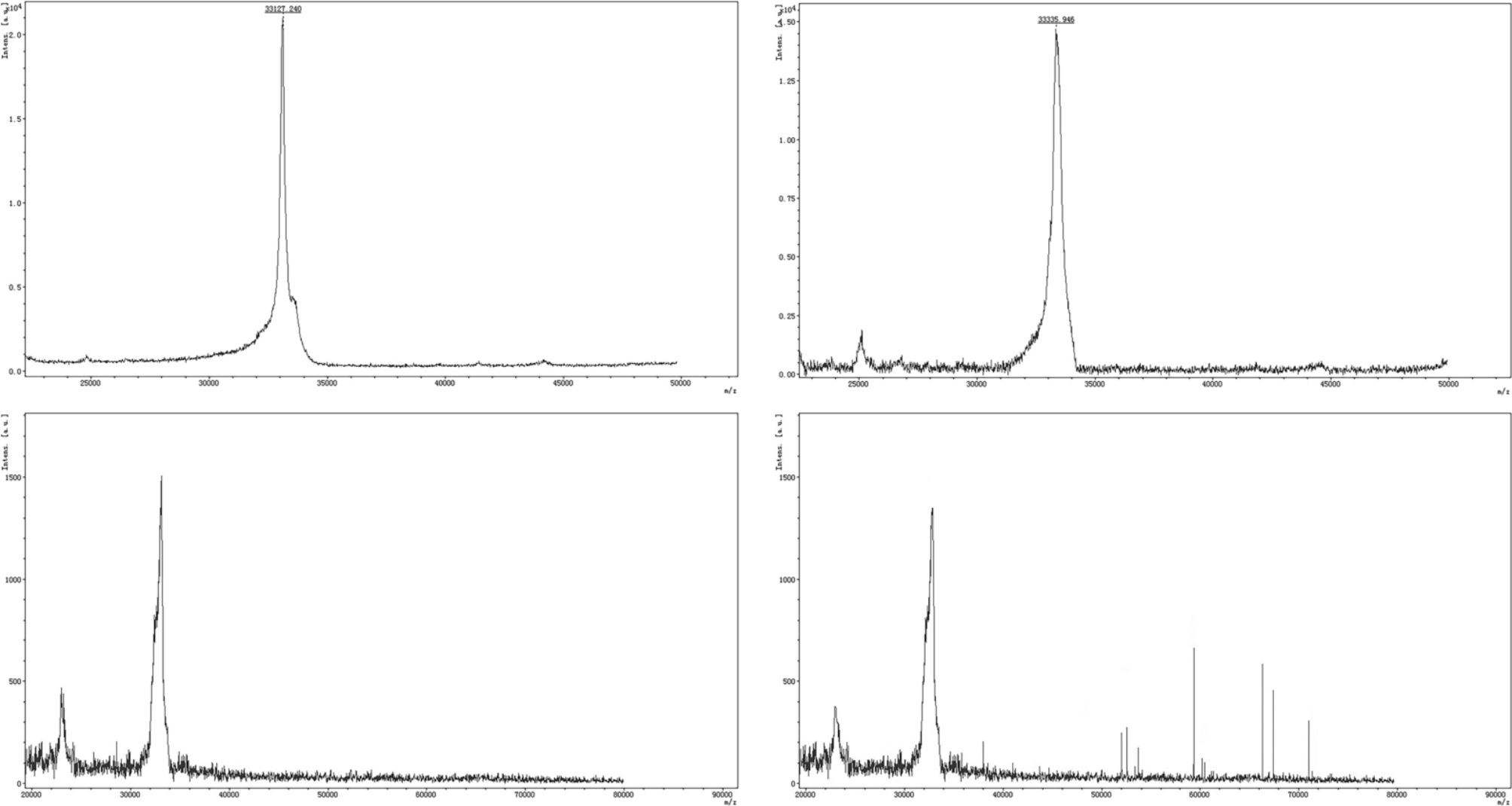

Supplement: Supplementary file 1 — Figure S1 Supporting Information [file JBM-110-1774-s001.tif]
