# Supplementary material for: Recombinant collagen hydrogels induced by disulfide bonds
Source: J Biomed Mater Res A. 2022 Jul 14;110(11):1774–85. doi: 10.1002/jbm.a.37427 (PMC9544300; doi:10.1002/jbm.a.37427)
Supplement: Supplementary file 2 — Supplemental Table 1 The designed sequence of VCL and S‐VCL‐S [file JBM-110-1774-s003.docx]

Supplemental Table 1. The designed sequence of VCL and S-VCL-S

| No. | Name of sequence | The designed sequence | The Optimized(for Escherichia coli)sequence |
| --- | --- | --- | --- |
| 1 | VCL | CCATGGGCHHHHHHADEQEEKAKVRTELIQELAQGLGGIEKKNFPTLGDEDLDHTYMTKLLTYLQEREQAENSWRKRLLKGIQDHALDLVPRGSGQDGRNGERGEQGPTGPTGPAGPRGLQGLQGLQGERGEQGPTGPAGPRGLQGERGEQGPTGLAGKAGEAGAKGETGPAGPQGPRGEQGPQGLPGKDGEAGAQGPAGPMGFPGERGEKGEPGTQGAKGDRGETGPVGPRGERGEAGPAGKDGERGPVGPAGKDGQNGQDGLPGKDGKDGQNGKDGLPGKDGKDGQNGKDGLPGKDGKDGQDGKDGLPGKDGKDGLPGKDGKDGQPGKPTAAGGATCC | CCATGGGCCACCACCACCACCATCATGCAGACGAACAAGAAGAGAAGGCCAAAGTTCGCACCGAGCTGATTCAAGAACTGGCCCAAGGTCTGGGCGGTATCGAGAAGAAGAACTTTCCGACTTTAGGCGATGAGGATTTAGACCATACCTACATGACCAAACTGCTGACCTATCTGCAAGAACGCGAGCAAGCTGAAAACAGCTGGCGCAAACGTCTGCTGAAAGGTATTCAAGATCACGCTTTAGATCTGGTGCCGCGTGGCAGTGGTCAAGATGGCCGCAATGGTGAACGTGGTGAACAAGGTCCTACCGGTCCGACTGGTCCCGCTGGTCCTCGTGGTCTGCAAGGTTTACAAGGTCTGCAAGGTGAACGCGGCGAACAAGGTCCGACTGGTCCCGCTGGTCCCCGTGGTTTACAAGGCGAGCGCGGTGAACAAGGTCCGACTGGTCTGGCTGGTAAAGCTGGTGAAGCTGGTGCCAAAGGCGAAACCGGCCCCGCTGGTCCTCAAGGTCCTCGCGGTGAGCAAGGTCCGCAAGGTTTACCGGGTAAAGATGGTGAGGCTGGTGCACAAGGCCCCGCTGGTCCGATGGGTTTCCCGGGTGAACGTGGCGAAAAAGGTGAACCGGGTACCCAAGGTGCCAAAGGTGATCGCGGTGAAACTGGTCCGGTGGGTCCCCGCGGCGAACGCGGTGAAGCCGGTCCCGCTGGCAAAGACGGTGAGCGTGGTCCGGTTGGCCCGGCTGGTAAAGACGGCCAGAATGGCCAAGATGGTCTGCCCGGTAAGGACGGCAAGGACGGCCAGAACGGCAAGGATGGCTTACCCGGTAAAGACGGCAAAGATGGTCAGAATGGCAAGGACGGTTTACCCGGCAAGGACGGTAAGGACGGCCAAGATGGCAAAGATGGTTTACCGGGTAAAGACGGCAAGGATGGCTTACCGGGCAAGGATGGTAAGGATGGTCAGCCCGGTAAACCGTAAGGATCC |
| 2 | S-VCL-S | CCATGGGCHHHHHH**C**ADEQEEKAKVRTELIQELAQGLGGIEKKNFPTLGDEDLDHTYMTKLLTYLQEREQAENSWRKRLLKGIQDHALDLVPRGSGQDGRNGERGEQGPTGPTGPAGPRGLQGLQGLQGERGEQGPTGPAGPRGLQGERGEQGPTGLAGKAGEAGAKGETGPAGPQGPRGEQGPQGLPGKDGEAGAQGPAGPMGFPGERGEKGEPGTQGAKGDRGETGPVGPRGERGEAGPAGKDGERGPVGPAGKDGQNGQDGLPGKDGKDGQNGKDGLPGKDGKDGQNGKDGLPGKDGKDGQDGKDGLPGKDGKDGLPGKDGKDGQPGKP**C**TAAGGATCC | CCATGGGCCACCATCATCACCATCACTGCGCAGATGAACAAGAAGAGAAGGCCAAGGTTCGCACCGAGCTGATTCAAGAACTGGCCCAAGGTCTGGGTGGCATCGAGAAGAAGAATTTCCCGACTTTAGGTGATGAAGATTTAGACCACACCTACATGACCAAGCTGCTGACATATTTACAAGAACGTGAACAAGCTGAAAACAGCTGGCGCAAACGTTTACTGAAAGGCATTCAAGATCATGCTTTAGATCTGGTTCCGCGTGGCTCTGGTCAAGATGGTCGTAACGGTGAACGCGGTGAACAAGGTCCTACTGGTCCGACCGGCCCGGCTGGTCCGCGTGGCTTACAAGGTTTACAAGGTTTACAAGGTGAGCGCGGCGAACAAGGTCCTACCGGTCCCGCTGGTCCTCGCGGTTTACAAGGCGAGCGTGGTGAGCAAGGTCCGACCGGTCTGGCCGGTAAAGCTGGTGAAGCTGGTGCCAAAGGTGAGACTGGCCCCGCTGGTCCGCAAGGTCCTCGTGGCGAGCAAGGTCCGCAAGGTTTACCCGGCAAAGACGGCGAGGCTGGCGCACAAGGTCCCGCTGGTCCTATGGGTTTTCCCGGTGAGCGCGGTGAGAAAGGTGAACCGGGCACCCAAGGTGCCAAAGGCGATCGTGGCGAAACCGGTCCCGTTGGTCCGCGTGGTGAACGTGGTGAGGCCGGCCCCGCCGGCAAGGATGGTGAGCGTGGCCCCGTTGGTCCGGCTGGCAAGGATGGCCAGAATGGCCAAGATGGTTTACCGGGTAAAGATGGCAAGGATGGCCAAAATGGTAAGGACGGCTTACCCGGCAAGGACGGTAAGGACGGCCAGAACGGCAAGGATGGTCTGCCGGGCAAGGACGGTAAAGACGGTCAAGACGGCAAAGACGGCTTGCCGGGCAAGGATGGTAAAGACGGCTTGCCCGGCAAAGACGGCAAGGACGGCCAGCCGGGCAAACCGTGCTAAGGATCC |
