# Supplementary material for: Recombinant collagen hydrogels induced by disulfide bonds
Source: J Biomed Mater Res A. 2022 Jul 14;110(11):1774–85. doi: 10.1002/jbm.a.37427 (PMC9544300; doi:10.1002/jbm.a.37427)
Supplement: Supplementary file 3 — Supplemental Table 2 The theoretical and measured molecular weight of protein [file JBM-110-1774-s002.docx]

Supplemental Table 2. The theoretical and measured molecular weight of protein

| Name | Length of protein | Theoretical Mw (kDa) | Measured Mw (kDa) | Error (%) |
| --- | --- | --- | --- | --- |
| VCL | 986 bp | 33.14 | 33.127 | 0.039 |
| S-VCL-C | 992 bp | 33.35 | 33.336 | 0.042 |
