# Supplementary material for: Recombinant collagen hydrogels induced by disulfide bonds
Source: J Biomed Mater Res A. 2022 Jul 14;110(11):1774–85. doi: 10.1002/jbm.a.37427 (PMC9544300; doi:10.1002/jbm.a.37427)
Supplement: Supplementary file 4 — Supplemental Table 3 Comparison of cell viability at 1 day among two experimental groups and the control group Supplemental Table 4. Comparison of cell viability at 1‐, 3‐, 7‐, and 14 days in experimental groups [file JBM-110-1774-s004.doc]

Supplemental Table 3. Comparison of cell viability at 1 day among two experimental groups and the control group

| Groups | Time | N | Mean | ±SD |  |
| --- | --- | --- | --- | --- | --- |
| PBS | 24h | 3 | 97.23 | ±1.89^bc^ |  |
| VCL | 24h | 3 | 43.32 | ±1.83^ac^ |  |
| S-VCL-S | 24h | 3 | 69.15 | ±1.55^ab^ |  |
|  | *F* value |  |  | 1287.493 |  |
|  | *P* value |  |  | *P*<0.05 |  |
| The cell viability of the two experimental and control groups was significantly different (*P*<0.05). The results of pairwise comparisons between groups are shown in the table. Compared with control group, ^a^ *P*<0.05; compared with VCL group, ^b^ *P*<0.05; compared with S-VCL-S group, ^c^ *P*<0.05. | | | | | |

Supplemental Table 4. Comparison of cell viability at 1-,3-,7- and 14 days in experimental groups

| Time | N | Mean | ±SD |
| --- | --- | --- | --- |
| 1 day | 3 | 18.23 | ±1.17^bcd^ |
| 3 days | 3 | 25.42 | ±1.75^acd^ |
| 7 days | 3 | 58.61 | ±1.61^abd^ |
| 14 days | 3 | 78.66 | ±2.12^abc^ |
| *F* value |  |  | 837.455 |
| *P* value |  |  | *P*<0.05 |
| There were significant differences in the cell viability among the experimental groups and the control groups at 1,3,7 and 14 days (*P* < 0.05). Compared with day 1, ^a^ *P*<0.05; compared with day 3, ^b^ *P*<0.05; compared with day 7, ^c^ *P*<0.05; compared with day 14, ^d^ *P*<0.05. | | | |
